# Recommendation of Early Surgery in Primary Mitral Regurgitation: Pros and Cons

**DOI:** 10.5935/abc.20160107

**Published:** 2016-08

**Authors:** Vitor Emer Egypto Rosa, João Ricardo Cordeiro Fernandes, Antonio Sergio de Santis Andrade Lopes, Tarso Augusto Duenhas Accorsi, Flavio Tarasoutchi

**Affiliations:** Instituto do Coração do Hospital das Clínicas da Faculdade de Medicina da Universidade de São Paulo, SP - Brazil

**Keywords:** Mitral Valve Insufficiency/surgery, Mitral Valve Prolapse, Heart Valve Prosthesis Implantation, Rheumatic Fever

Primary mitral valve regurgitation is the most frequent valvular pathology, affecting approximately 1.7% of the population, with prolapsed mitral as the highlighted etiology.^1^ In developing countries, such as Brazil, rheumatic fever is still widely prevalent (18.6/1000), and affects about 15.6 million people worldwide.^2,3^ Currently, the recommendation of mitral surgery for asymptomatic patients is very controversial, since the indication of valvular intervention for symptoms, left ventricular disfunction and dilatation, recent onset atrial fibrillation or pulmonary arterial hypertension is well consolidated in literature (Table 1).^4-6^ One strand defends the concept of "*watchful waiting*", highlighting operative risks and morbi-mortality in early implantation of a bioprosthesis, as well as the benign history of asymptomatic mitral regurgitation. On the other hand, a second group advocates for the indication of early surgery, showing, through literature, that mitral valve repair, in these conditions, reduces surgical mortality and increases survival for these patients.^7-11^

**Table 1:** Recommendations for surgical treatment of primary mitral regurgitation

	**AHA^4^**	**ESC^5^**	**SBC^6^**
Symptomatic patients with LVEF >30%.	I B	I B	I B
Symptomatic patients with LV dysfunction (LVEF 30%-60% and/or LVSD ≥ 40 mm).	I B	I C LVSD ≥ 45 mm	I B
		IIa C LVSD ≥ 40 mm if repair	
Asymptomatic patients, non-rheumatic, with preserved LVEF and recent onset AF or pulmonary hypertension (SPAP > 50).	IIa B	IIa C	IIa C
Repair in asymptomatic patient with LVEF > 60% e LVSD < 40 mm with an estimated success rate of mitral valve repair > 95% and operative risk < 1%, in a reference center.	IIa B	IIb C Left atrium dilatation (volume ≥ 60 ml/m^2^) and sinus rhythm or pulmonary hypertension during exercise (SPAP ≥ 60 mmHg)	IIa B
Symptomatic patients with LVEF ≤ 30%, under optimized medication therapy	IIb C	IIa C Repair	IIb C
		IIb C Surgery	
Repair in rheumatic patients with estimated success rate of mitral valve repair > 95% or if anticoagulation reliability is questionable.	IIb B		IIb B

LVEF: left ventricular ejection fraction; LVSD: left ventricular systolic diameter; SPAP: systolic pulmonary arterial pressure; AHA: American Heart Association; ESC: European Society of Cardiology; SDB: Sociedade Brasileira de Cardiologia; AF: atrial fibrillation

A pivotal fact for the indication of early surgery is the possibility of effective mitral valve repair, considering early surgey with bioprosthesis implantation would bring the disadvantage of future operations and complications related to the prosthesis, while mechanical prosthesis implantation, due to high risk of thrombosis, would have the inconvenience of oral anticoagulation drug intake with warfarin indefinetely.

The natural history of rheumatic mitral regurgitation, which affects younger people than degenerative valves, associated to valvular destruction, commissural fusion, fusion of chordae tendineae, retraction, fibrosis, and calcification of the cusps, makes it difficult to perform a mitral valve repair, and difficult to indicate early surgery for this subgroup of patients.^12^ However, with regards to prolapsed mitral, patients with P2 segment prolapse undergoing surgical treatment in reference centers with high success rates in mitral valve repair (over 95%) have a high probability of reaching a good result with the procedure. Even though success rate may be lower depending on the complexity of the lesion and number of affected scallops, there is evidence that this intervention is feasible for all types of prolapses.^13^

Four authores support early surgery in low operative risk patients (under 1%). Suri et al.^7^ described MIDA results (*Mitral Regurgitation International Database registry*), a registry with 6 centers, 1021 patients between 1980 and 2004, showing higher 10-year survival with early surgery (86% vs 69%, p < 0.001). However, some biases are found, especially due to the time the study began (1980), patients with Class IIa surgical recommendations were included (10% with atrial fibrillation and 11.8% with pulmonary arterial hypertension). Moreover, it was a retrospective study, and *"watchful waiting"* was not ideal (each patient did the segment according to his/her doctor). Kang et al.^9^, in their first study, showed a higher 7-year survival rate with early surgery (99 ± 1% versus 85 ± 4%, p = 0.007). In the study, although the conservative group segment was inadequate, only three sudden deaths occurred (1.04%) in asymptomatic patients without markers of cardiac dysadaptation, showing a benign natural history in this group of patients. Using the same registry and adding one more center, five years later, Kang et al.^10^ published new work demonstrating cardiac mortality reduction with early surgery (5 ± 2% vs 1 ± 1%, p = 0.016). However, events that are possibly related to valvar surgery, such as stroke, were not counted. In these three studies,^7,9,10^ cardiac events were more frequent in the watchful waiting group, which was expected since this strategy's approach is to wait for symptoms or echocardiographic alterations for intervention recommendation. Montant et al,^8^ also in a retrospective, non-randomized study, showed better 10-year survival with the early surgery strategy (86% ± 4% vs 50% ± 7% p < 0.0001).

Enriquez-Sarano et al^11^ demonstrated that, in asymptomatic patients without risk markers, those with effective regurgitant orifice (ERO) ≥ 40 mm² showed higher mortality in comparison to those with ERO between 39-20 and under 20 (30 ± 9% vs 20 ± 6% vs 3 ± 2%, p < 0.01). This pointed to the existence of subgroups of patients who benefited from early surgery depending on markers that were not contemplated by guidelines.

Another subclinical dysfunction marker of the left ventricle is the brain natriuretic peptide (BNP). Although plasma levels are lower when compared to non-valvular heart failure, BNP increase is associated to mortality in asymptomatic mitral regurgitation patients.^14^ However, the limits that refer the patients to interventional treatment have not yet been defined.

The work of Rosenhek et al.^15^ was the only one in favor of watchful waiting. They describe an 8-year survival in 91 ± 3%, but there was no comparative group submitted to early surgery. There is only one meta-analysis on the subject, which only includes the five aforementioned studies that describe a possible advantage of the early surgery strategy. Nevertheless, the power of meta-analysis is limited by the power of the evaluated studies.^16^

Therefore, when assessing the pros and cons (Table 2), we believe that the strategy should be individualized and supported by the *Heart Team* decision. In our institution, the *Heart Team* consists of clinical professionals specialized in valvular diseases, experts in imaging diagnosis (echocardiography, cat-scan and cardiac MRI), a cardiothoracic surgeon and a hemodynamicist, all experienced in these specific cardiology areas, who contribute to treatment decisions in complex cases. In view of technology and communication advances, such group may be structured and meet remotely.

**Table 2:** Pros and cons of early surgery

**Pros of *early surgery***	**Cons of *early surgery***
Isolated mitral prolapse of the P2 segment	Prolapse with complex morphology or rheumatic mitral regurgitation
Mortality reduction according to Suri et al.,^7^ Montant et al.^8^ e Kang et al.^9,10^	Publication biases - non-randomized retrospective works^7-10^
Existence of prognostic factors not contamplated in the guidellines (ERO and BNP)^11-14^	Benign natural history of asymptomatic mitral regurgitation^15^
Reference center in valvulopathies with estimated success rate in mitral valve repairs > 95%^4-6^	Centers with few realized repairs

ERO: effective regurgitant orifice; BNP: brain natriuretic peptide.

In this moment, in our country, the indication of early surgery is almost an exception, since it depends on the patient's characteristics (young, low operative risk, isolated mitral prolapse of the P2 segment) and on hospital structure. Such structure ranges from the echocardiography doctor's experience (valvular lesion complexity assessment) to the ability of the surgical team (high rates of mitral valve repairs) and postoperative care in specialized ICUs and infirmary. Moreover, possible risks must be exposed to the patient and his/her family. This indication would have the advantage of the survival benefit, validated by the evidence in literature, and the disadvantage, if the mitral valve repair is not successful,^7-11,16^ of premature bioprosthesis implant. If the patient or surgical service do not qualify, watchful waiting would seem the more adequate strategy. However, despite its name, the conservative treatment is "aggressive". The patient must undergo periodical clinical echocardiographic evaluation (preferably every 6 months, assessing trends in echocardiographic values), be instructed to seek medical help if symptoms appear and, most importantly, be referred to surgery, without delays, as soon as any criterion indicative of intervention is met.
